# Decrease in walking ability with increased functional connectivity between multiple brain areas in Parkinson’s disease: a functional near-infrared spectroscopy study

**DOI:** 10.3389/fnagi.2024.1454598

**Published:** 2024-10-30

**Authors:** Jin Wang, Jiewei Lu, Yue Wang, Zhilin Shu, Yuanyuan Cheng, Xinyuan Zhang, Yang Yu, Jianda Han, Zhizhong Zhu, Ningbo Yu, Jialing Wu

**Affiliations:** ^1^Clinical College of Neurology, Neurosurgery and Neurorehabilitation, Tianjin Medical University, Tianjin, China; ^2^Department of Neurology, Tianjin Huanhu Hospital, Tianjin, China; ^3^College of Artificial Intelligence, Nankai University, Tianjin, China; ^4^Department of Rehabilitation Medicine, Tianjin Huanhu Hospital, Tianjin, China; ^5^Tianjin Key Laboratory of Cerebral Vascular and Neurodegenerative Diseases, Tianjin Neurosurgical Institute, Tianjin, China

**Keywords:** fNIRS, Parkinson’s disease, functional connectivity, premotor cortex, gait

## Abstract

**Introduction:**

Gait disturbances significantly impact the mobility and quality of life of individuals with Parkinson’s disease (PD). This study aims to delve into the cortical mechanisms underlying gait disorders in PD, specifically focusing on the prefrontal cortex (PFC), premotor cortex (PMC), and primary somatosensory cortex (PSC).

**Objective:**

To compare the functional connectivity of the PFC, PMC, and PSC regions during walking between individuals with PD and healthy controls.

**Methods:**

The study included 30 individuals with PD (mean age 62.40 ± 7.16 years) and 22 healthy older adults (mean age 60.95 ± 6.34 years). All participants were requested to walk back and forth at a comfortable pace for 30 s over a 10-meter course three times. A mobile functional near-infrared spectroscopy (fNIRS) system was employed to evaluate the oxyhemoglobin concentration change (∆HbO2). To assess the interactions between the PFC, PMC, and PSC, the connectivity strength between different fNIRS channels was computed.

**Results:**

Individuals with PD in the off-state exhibited significantly decreased walking speed and shorter stride length compared to the healthy controls. For six brain regions including the left (L) and right (R) PFC, PMC, and PSC, no significant differences in functional connectivity within each region were found between the PD and control groups. However, when it comes to the functional connectivity between every two regions, the PD group exhibited stronger functional connectivity than the control group in the LPFC-LPMC, LPFC-RPMC, LPFC-LPSC, RPFC-LPMC, RPFC-LPSC, LPMC-LPSC, LPMC-RPSC, and RPMC-RPSC. Positive correlations were found between gait performance (speed and stride length) and functional connectivity within the RPMC as well as between the RPMC and the RPSC.

**Conclusion:**

Individuals with PD exhibit notable gait disturbances and increased functional connectivity in brain regions responsible for sensorimotor integration and motor function in their off-state. Strengthening the functional connectivity within the RPMC and between the RPMC and the RPSC could be a potential target for future treatments of gait impairments in PD.

## 1 Introduction

Gait disturbance, which is a core motor symptom in Parkinson’s disease (PD), may have a significant impact on patients’ mobility and quality of life (Wei-Shan et al., 2021). As we know, the formation and control of gait are inseparable from the information connections among key brain regions, such as the frontoparietal cortex, basal ganglia area and cerebellum, etc. (Weiss et al., 2020; Camargo et al., 2024). In functional imaging, the functional connectivity (FC) of relevant brain regions is a good and direct quantitative indicator for reflecting this communication (Friston, 2011). Previous studies have shown that PD can be regarded as a neural network disease. For example, PD patients with freezing of gait (PD-FOG) exhibited increased FC between the visual network and the left caudate nucleus (Kenan et al., 2021), and the FC between the left putamen, retrosplenial cortex, and cerebellum was also increased (Alexandra et al., 2019). Furthermore, PD-FOG displayed a higher within-network FC and reduced FC between right frontoparietal and executive-control networks (Komal et al., 2019). Previous functional magnetic resonance imaging (fMRI) studies mentioned above have explored FC change in PD-FOG, but the results remain inconsistent (Kim and Fraser, 2022). Besides, to our knowledge, the characteristics of brain FC in PD patients during walking in a real environment, especially in the frontoparietal cortex have not been fully studied.

Functional near-infrared spectroscopy (fNIRS) is a technique that relies on blood-oxygen-level-dependent (BOLD) signals. fNIRS quantifies hemodynamic responses associated with neuronal activation. (Rahman et al., 2020). It offers several major advantages, including lightweight, and wearability. fNIRS has been applied to investigate brain activity in a real environment during various walking paradigms in PD patients (Nieuwhof et al., 2016; Stuart et al., 2018). The prefrontal cortex (PFC) plays a crucial role in walking and dual tasks (Nutt et al., 2011; Tachibana et al., 2012). Previous studies revealed that PD patients showed greater PFC activation than matched controls (Maidan et al., 2015; Maidan et al., 2016). PD patients with postural instability gait disorder presented a higher PFC activity compared with tremor-dominant patients during walking (Orcioli-Silva et al., 2021a). The latest research has confirmed that PD-FOG patients exhibited greater PFC activation during both single and dual-task walking than PD patients without FOG (PD-nFOG) (Vitorio et al., 2020). The increased brain activity might compensate for motor and cognitive impairments in people with PD (Nieuwhof et al., 2017). The prefrontal cortex plays a crucial role in gait control in PD patients, but the premotor cortex and primary somatosensory cortex are also involved.

The premotor cortex (PMC) and primary somatosensory cortex (PSC) play a pivotal role in the basal-ganglia cortical loop (Nachev et al., 2008). PSC excitability could be involved in changes in somatosensory integration in PD (Palomar et al., 2011). A study has shown that PD medication increases sensorimotor integration during walking by increasing the posterior parietal cortex (CPz) activity (Orcioli-Silva et al., 2021b). fNIRS has been applied to assess FC changes in neurodegenerative disorder groups (Wang et al., 2022). The FC between PFC and sensorimotor cortex (SMC) of PD-FOG patients was higher during normal stepping than that of PD-nFOG patients and healthy controls (HCs) (HongSheng et al., 2023). Our previous fNIRS-based FC study demonstrated that FC analysis of the PFC, parietal lobe, and occipital lobe can optimize deep brain stimulation (DBS) Stimulation parameters (Ningbo et al., 2021). In addition, we proposed an fNIRS-based brain connectivity state which is significantly correlated with gait performance (Jiewei et al., 2023). However, in individuals with PD, the FC of the PFC, PMCand PSC, and interaction among these regions during walking in the “off-state” remains poorly understood.

In this study, we applied wearable fNIRS devices to measure the FC of PFC, PMC, and PSC, and its association with gait performance in HCs and PD patients. The purpose of this study was to quantify changes in brain FC interaction between the PFC, PMC, and PSC involved in the walking paradigm. We hypothesized that PD patients have hyperconnected networks within and between the cortical regions responsible for gait executive (PFC), sensorimotor integration (PSC), and motor function (PMC).

## 2 Materials and methods

### 2.1 Participants

This study was approved by the Tianjin Huanhu Hospital Ethics Committee (NO.2019-31) and conducted following the principles of the Declaration of Helsinki. This study was registered in the Chinese Clinical Trial Registry as “Analysis of related factors of function of participants with Parkinson’s disease” (the registration number is ChiCTR1900022655; Date of Registration: 2019-04-20, https://www.chictr.org.cn/showproj.html?proj=38201). All participants provided written informed consent for participation in the study. A total of 30 consecutive inpatient and outpatient PD patients from the Department of Neurology, Neurosurgery, and Rehabilitation at the Tianjin Huanhu Hospital and 22 healthy adults were recruited between March 2020 and March 2022. Inclusion criteria: (1) Clinically established PD patients diagnosed by an experienced neurologist according to the Movement Disorder Society’s (MDS) diagnostic criteria (Postuma et al., 2015). (2) The patients should have the ability to walk independently for 30 meters before taking anti-Parkinson medications without any assistive devices. (3) Mild to moderate stages of the Hoehn and Yahr scale (≤3; Hoehn and Yahr, 1967). (4) All participants demonstrated normal cognitive function (MMSE>24). Exclusion criteria: (1) Participants with arthritis, cardiovascular disease, or other neurological disorders that hinder the completion of the walking test were excluded from the study; (2) comorbidities that were likely to affect gait; (3) history of DBS surgery. All assessments were conducted while the PD participants were in the “off-state” of medication including the Movement Disorders Society Unified Parkinson’s Disease Rating Scale, motor part (MDS-UPDRS III; Goetz et al., 2008), gait analysis, etc. “Off-state” is defined as withholding all dopaminergic medications for a minimum of 12 h, all assessments were done early in the morning before taking the pills. The sample size was calculated using G*Power v3.1.9. The estimated sample size of the PD group of 26 patients was enough. Considering possible missing data, a total of 30 PD participants were recruited for this study.

### 2.2 Procedures

A mobile and portable fNIRS system (Nirsmart, Danyang Huichuang Medical Equipment Co, Ltd., China) was used to record changes in oxygenated hemoglobin (HbO2) and deoxygenated hemoglobin. A wireless APDM Movement Monitoring inertial sensor system (Mobility Lab, APDM Inc., USA) was used to record gait performance. Signals were sampled in accordance with a previous study (Lanovaz et al., 2017).

For the walking task, participants were requested to walk back and forth at a comfortable pace for 35 s over a 10 m-long course on a firm surface in our rehabilitation center. Each end of the course was marked to indicate when the participants should make an arc-shaped turn. The walking tasks were performed 3 times and between each trial with 2-min blocks. Each task started with 30 s of standing still, followed by 35 s of walking, and another 10 s of standing to calm down.

**Figure 1:**
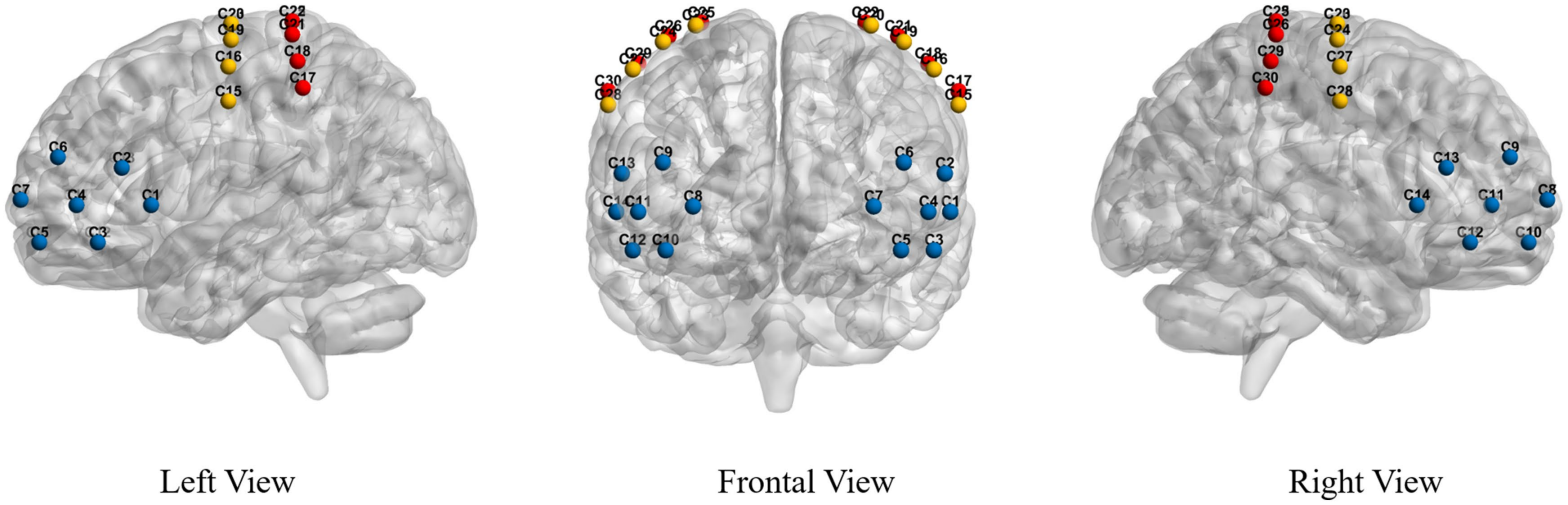
Position of the fNIRS channels.

Functional Near Infrared Spectroscopy: The fNIRS system used a near-infrared light of 730 nm and 850 nm to measure the optical intensities for HbO2, with a sampling rate of 11 Hz. A total of 26 fNIRS probes, including 14 sources and 12 detectors, were placed within and between regions of interest (ROIs). Probes were placed over the PFC, PMC, and PSC according to the 10–20 International System of Electrode Placement. Probes provide a total of 30 fNIRS channels covering all the ROIs responsible for gait control in this study, see Figure 1. The source-detector distance was 3 cm.

### 2.3 Data analysis

We detailed this section: Preprocessing was performed with the following steps: (1) Motion artifacts were identified using a threshold-based approach and corrected by cubic spline interpolation. (2) A bandpass filter ranging from 0.01 to 0.2 Hz was employed to eliminate physiological noise, such as heartbeats and respirations. (3) Changes in oxyhemoglobin concentrations (ΔHbO) were calculated using the modified Beer–Lambert law. (4) Principal component analysis was carried out to eliminate systemic physiological fluctuations such as scalp blood flow interference. (5) The processed signals during the 0–35-s walking period were extracted and corrected for a 5-s pre-walk baseline interval for detailed analysis. Preprocessing was implemented by the NirSpark software (version 1.6.1, Danyang Huichuang Medical Equipment Co., Ltd., China) and Matlab R2021a.

In this paper, FC was measured to analyze the preprocessed brain signals. Specifically, the connectivity strength between different fNIRS channels was calculated with the Pearson correlation coefficient. Subsequently, the connectivity strength was averaged over brain regions and all trials to obtain the final FC strength, which was used to analyze the interactions between different brain regions.

### 2.4 Statistical analysis

Statistical analysis was performed using SPSS 25.0 for Windows. The normality of data was tested by the Shapiro–Wilk test and variance homogeneity by the Levene test. The Mann–Whitney U test was used to evaluate differences in the demographic data between the PD group and the HCs group. Gender differences between groups were evaluated using the Chi-squared test. Data not conforming to a normal distribution were compared by the Mann–Whitney U test and the Kruskal–Wallis test. The Kruskal–Wallis test was followed by Dunn-Bonferroni post-hoc tests to analyze differences between the groups. Two-sided paired t-tests were used to assess group differences. Pearson correlation analysis was employed to analyze the correlation between the FC, and gait performance. The significance level of all analyses was set at 5%.

## 3 Results

### 3.1 Characteristics of the participants

30 PD patients and 22 HCs matched by age and sex completed the experimental procedure. Table 1 presents all the participants’ demographic characteristics and clinical assessment scores. No significant difference was observed in age, sex, or cognitive scores between the two groups. The disease severity in the PD group ranged from mild to moderate (Hoehn and Yahr Scale staged I to III). The motor symptom scores (MDS-UPDRS III) showed a significant improvement after medication in PD patients (19.17 ± 8.47 vs. 32.80 ± 11.90, *p* < 0.001). The average levodopa equivalent daily dose of the PD patients’ dopaminergic medication was 502.46 ± 216.02 mg/day.

**Table 1:** Participant characteristics (Mean ± SD).

	PD (*n* = 30)	HCs (*n* = 22)	*p*-value	Cohen’s d effect size
Age (years)	62.40 ± 7.16	60.95 ± 6.34	0.455	0.21
Sex (male/female)	15/15	12/10	0.752	
Disease duration (years)	4.95 ± 2.96	/		
MMSE (0–30)	27.43 ± 1.97	27.09 ± 1.55	0.503	0.19
MDS-UPDRS III: off (0–132)	32.80 ± 11.90 @	/	**0.000@**	1.32
MDS-UPDRS III: on (0–132)	19.17 ± 8.47	/	/	
H&Y (I/II/III)	2/6/22	/	/	
LED (mg/d)	502.46 ± 216.02	/	/	

LED, levodopa equivalent daily dose; MDS-UPDRS III, Movement Disorders Society Unified Parkinson’s Disease Rating Scale, motor part; MMSE, Mini-Mental State Examination; H&Y, Hoehn and Yahr Scale; @ Difference between PD “off” and “on” state. @ *p* < 0.01, compared with on state.

### 3.2 Gait performance

Gait performance is shown in Table 2. The cadence, double support (%), and gait cycle durations showed no statistical difference between the two groups. In contrast, a statistically significant difference was observed in gait parameters, including gait speed (p < 0.01) and stride length (*p* < 0.01). Participants with PD walked more slowly and had shorter stride lengths compared with HCs.

**Table 2:** Gait parameters of the HCs group and PD group (Mean ± SD).

Gait parameters	PD (*n* = 30)	HCs (*n* = 22)	*p*-value	Cohen’s d effect size
Cadence L (step/min)	113.87 ± 10.68	113.01 ± 7.13	0.745	0.09
Cadence R (step/min)	114.02 ± 10.72	113.15 ± 7.11	0.743	0.09
Double support L (%)	21.36 ± 4.93	19.53 ± 3.06	0.131	0.43
Double support R (%)	21.41 ± 4.86	19.57 ± 3.06	0.126	0.44
Gait cycle duration L (s)	1.07 ± 0.10	1.07 ± 0.07	0.939	0.00
Gait cycle duration R (s)	1.06 ± 0.10	1.07 ± 0.07	0.903	−0.11
Gait speed L (m/s)	**0.97 ± 0.22**	**1.12 ± 0.15**	**0.007∗**	−0.77
Gait speed R (m/s)	**0.97 ± 0.21**	**1.12 ± 0.15**	**0.006∗**	−0.80
Stride length L (m)	**1.02 ± 0.21**	**1.19 ± 0.12**	**0.001∗**	−0.96
Stride length R (m)	**1.01 ± 0.20**	**1.18 ± 0.12**	**0.001∗**	−0.99

L, left side; right side. *p < 0.01, compared with healthy controls.

### 3.3 The FC within and between ROIs

**Figure 2:**
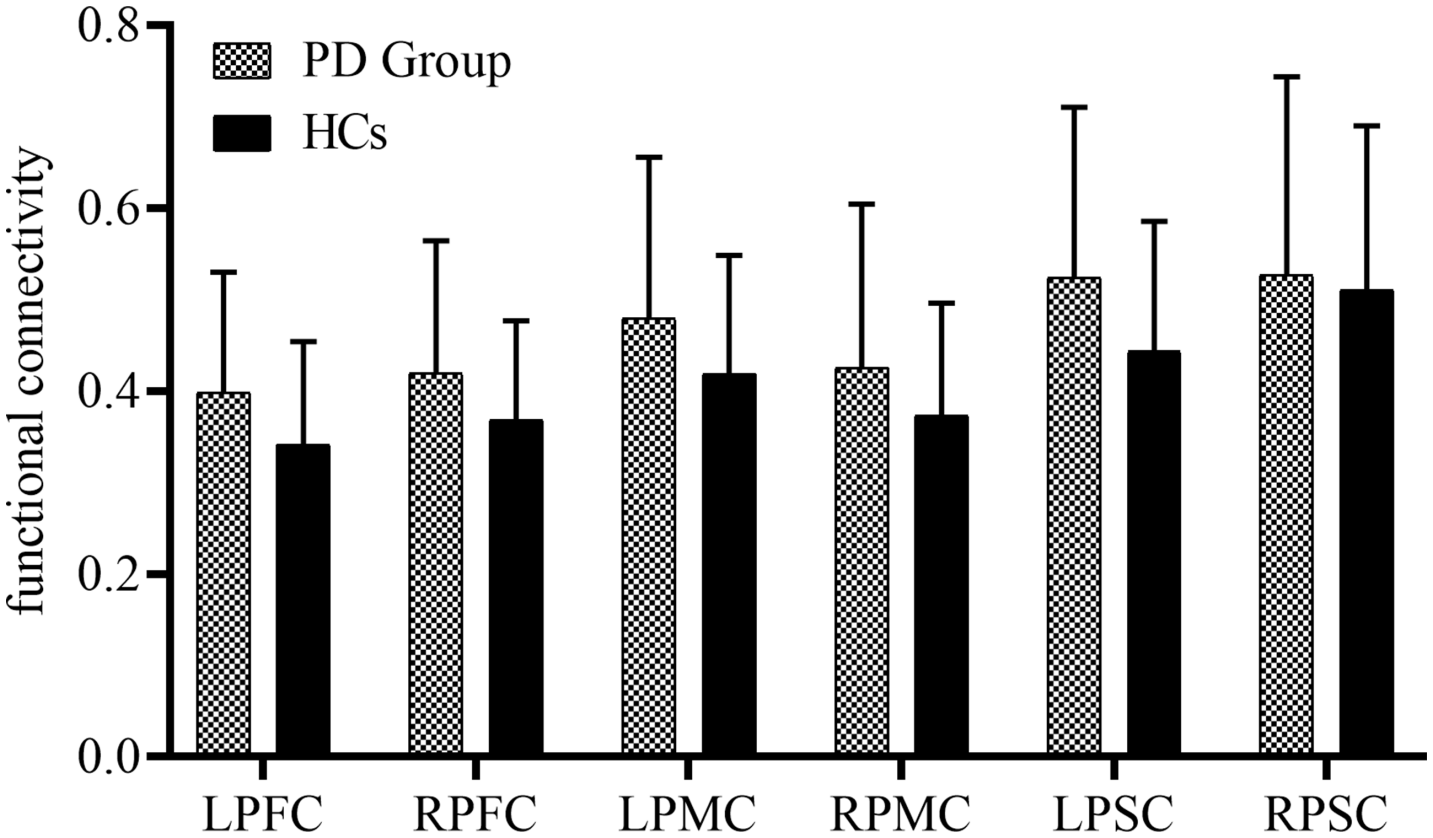
Summary of the functional connectivity differences between the study groups. PFC, prefrontal cortex; PMC, premotor cortex; PSC, primary somatosensory cortex; L, left side; right side.

**Figure 3:**
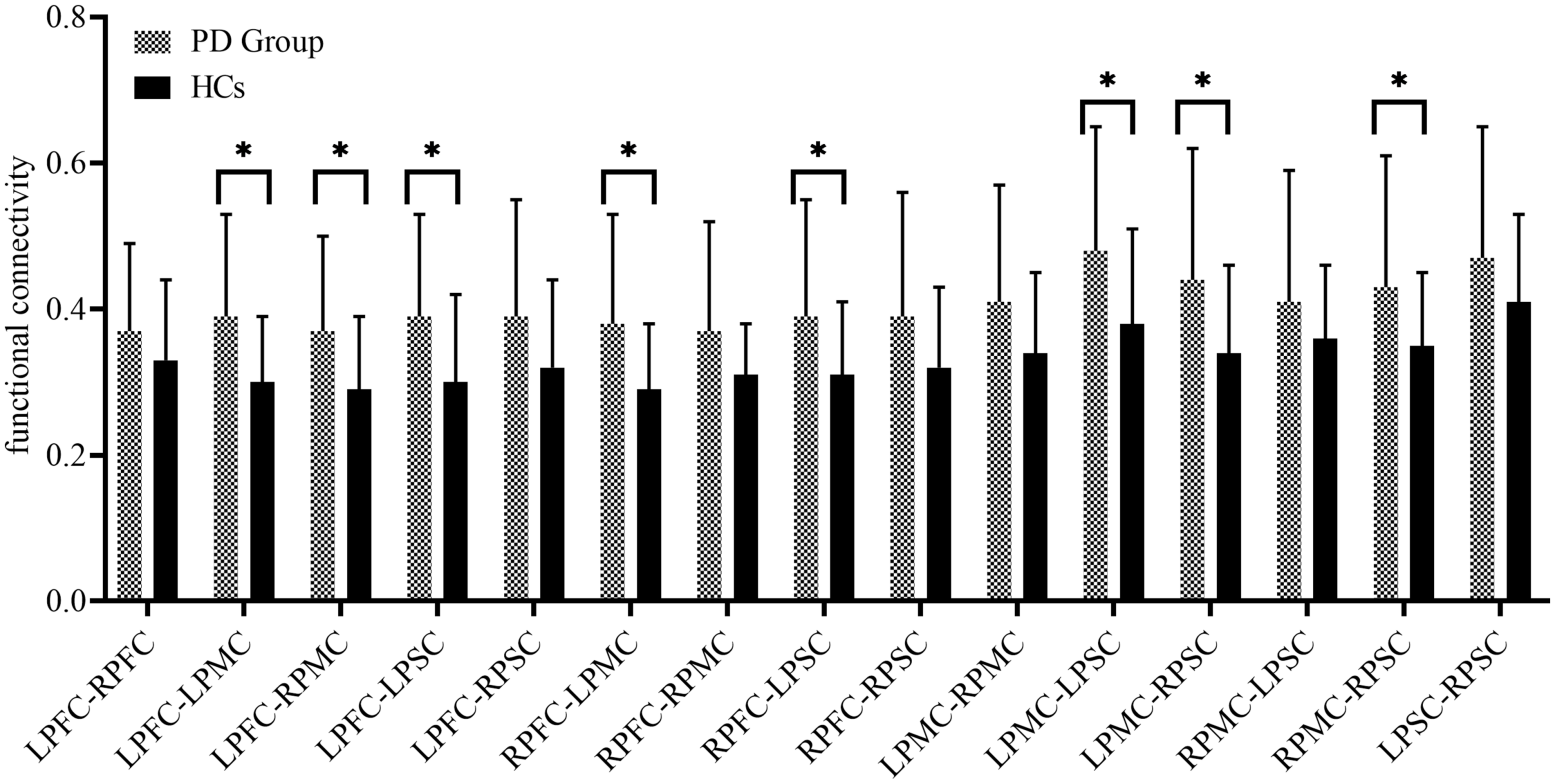
Summary of the functional connectivity between brain regions. PFC, prefrontal cortex; PMC, premotor cortex; PSC, primary somatosensory cortex; L, left side; right side.

Figure 2 presents the FC of ROIs for the 22 HCs and 30 PD patients during walking conditions. The PD group and HCs exhibited no statistical difference among the FC of ROIs. LPFC (0.40 ± 0.13 vs. 0.34 ± 0.11, *p* = 0.11), RPFC (0.42 ± 0.14vs. 0.37 ± 0.11, *p* = 0.17), LPMC (0.48 ± 0.18vs. 0.42 ± 0.13, *p* = 0.18), RPMC (0.43 ± 0.18vs. 0.37 ± 0.12, *p* = 0.22),LPSC (0.52 ± 0.19vs. 0.44 ± 0.14, *p* = 0.10), RPSC (0.53 ± 0.22vs. 0.51 ± 0.18, *p* = 0.78). The FC between ROIs for 22 HCs and 30 PD is represented in Figure 3. Compared with HCs, the PD group demonstrated significantly higher FC values during walking between LPFC-LPMC (0.39 ± 0.14 vs. 0.30 ± 0.09, *p* = 0.024, Cohen’s d effect size = 0.65), LPFC-RPMC (0.37 ± 0.13 vs. 0.29 ± 0.10, *p* = 0.032, Cohen’s d effect size = 0.62), LPFC-LPSC (0.39 ± 0.14 vs. 0.30 ± 0.12, *p* = 0.026, Cohen’s d effect size = 0.64), RPFC-LPMC (0.38 ± 0.15 vs. 0.29 ± 0.09, *p* = 0.01, Cohen’s d effect size = 0.75), RPFC-LPSC (0.39 ± 0.16 vs. 0.31 ± 0.10, *p* = 0.043, Cohen’s d effect size = 0.58), LPMC-LPSC (0.48 ± 0.17 vs. 0.38 ± 0.13, *p* = 0.022, Cohen’s d effect size = 0.66), LPMC-RPSC (0.44 ± 0.18 vs. 0.34 ± 0.12, *p* = 0.028, Cohen’s d effect size = 0.64), and RPMC-RPSC (0.43 ± 0.18 vs. 0.35 ± 0.10, *p* = 0.045, Cohen’s d effect size = 0.53).

### 3.4 Associations between FC coupling strength and behavioral performance

**Figure 4:**
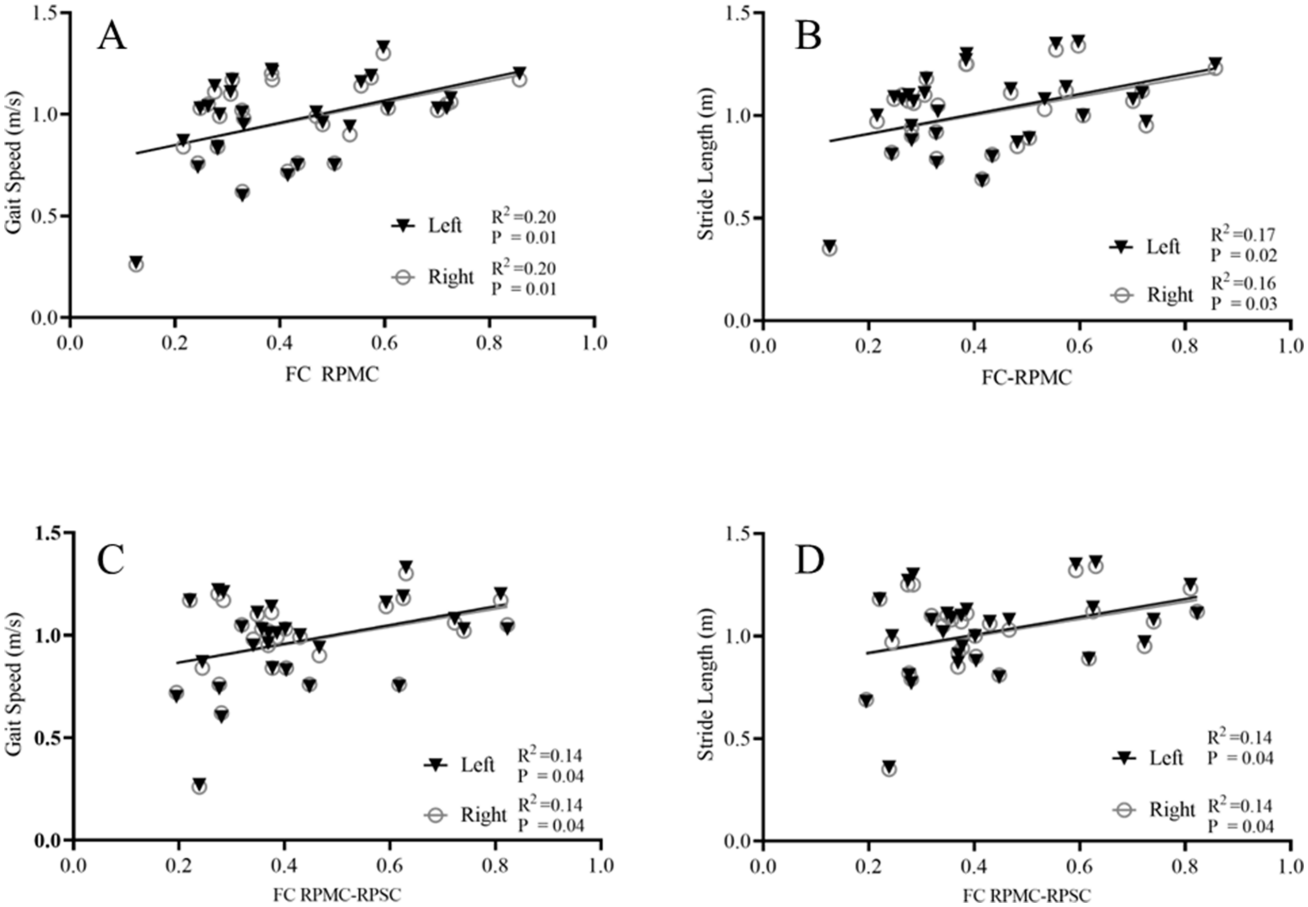
Correlation between FC coupling strength and gait parameters. Correlation analysis between the FC coupling strength and gait parameters. The FC coupling strength of the right PMC was positively correlated with the gait speed (A) and stride length (B); The FC coupling strength between RPMC-RPSC was positively correlated with the gait speed (C) and stride length (D). FC, functional connectivity; PMC, premotor cortex; PSC, primary somatosensory cortex; R, right side.

For the PD group, the FC coupling strength of RPMC was positively correlated with the gait speed (r = 0.445/0.443, R^2^ = 0.20/0.20, *p* = 0.01/0.01) and stride length (r = 0.411/0.411, R^2^ = 0.17/0.16, *p* = 0.02/0.03). In addition, the FC coupling strength between RPMC-RPSC was positively correlated with gait speed (r = 0.370/0.371, R^2^ = 0.14/0.14, *p* = 0.04/0.04) and stride length (r = 0.371/0.376, R^2^ = 0.14/0.14, *p* = 0.04/0.04), as displayed in Figure 4.

However, no correlation was found between the FC coupling strength of ROIs and gait parameters in the healthy control group. The FC coupling strength of RPMC was not correlated with the gait speed (r = 0.199/0.169, R^2^ = 0.04/0.03, *p* = 0.37/0.45) and stride length (r = 0.109/0.072, R^2^ = 0.01/0.01, *p* = 0.63/0.75). The FC coupling strength between RPMC-RPSC was not correlated with gait speed (r = 0.058/0.005, R^2^ = 0.00/0.00, *p* = 0.80/0.98) and stride length (r = 0.050/0.011, R^2^ = 0.00/0.00, *p* = 0.83/0.96).

## 4 Discussion

At present, few investigations have been carried out on the FC of specific brain regions in the cerebral cortex during a real walking state in patients with PD. Our study offers fNIRS-based evidence that, while performing a walking task, the FC between pairs of regions, like LPFC-LPMC, LPFC-RPMC, LPFC-LPSC, RPFC-LPMC, RPFC-LPSC, LPMC-LPSC, LPMC-RPSC, and RPMC-RPSC, is stronger in PD patients than in HCs. This study may be helpful for an in-depth understanding of the biological mechanisms of PD development from the perspective of neural networks.

Our results support previous findings that the cortices for motor control and sensory processing are more activated when the motor automaticity function is impaired. The present study also shows that, regarding the PFC, PMC, and PSC, there are no significant differences in the functional connections within each brain region or between the same brain regions of the bilateral hemispheres when comparing PD patients with normal individuals. In other words, the cortical network alterations in PD are mainly manifested as the coordinated changes among different brain regions.

Additionally, in our study, positive relations were found between gait performance (speed and stride length) and FC within the RPMC as well as between the RPMC and the RPSC. A previous resting-state fMRI study supports this. It shows that PD-FOG is associated with a decreased FC of the PMC and supplementary motor area bilaterally in the sensorimotor network, frontoparietal regions in the default mode network, and occipital cortex in the visual associative network (Elisa et al., 2015). So it seems that the enhanced FC in the key frontal–parietal areas might imply the result of compensation. This may be because firstly, existing evidence indicates that the abnormal gait of PD patients is mainly associated with the shortage of resources in the striatum, mitochondrial dysfunction in the cerebral cortex, as well as proprioceptive defects that are essential for balance (Ana et al., 2009; Sungjae et al., 2016; Kenan et al., 2021). Secondly, according to previous research, the firing of PMC correlates with multiple movement parameters, such as speed, acceleration, and position in individuals with PD. The sensorimotor network plays a crucial role in detecting and processing sensory input and in preparing and executing motor functions in PD (Alessandro et al., 2019). Thirdly, another study (Alexandra et al., 2019) shows that levodopa can enhance the FC of the sensorimotor network in the supplementary motor area also supports this. Therefore, our results may provide support for the formulation of rehabilitation strategies based on specific stimulation, and help evaluate the role of treatment in improving brain function.

There have been some previous studies on the brain FC in PD. For example, previous research by HongSheng et al. (2023) found that patients with PD and PD-FOG had stronger FC between PFC and sensorimotor cortex (SMC) compared to HCs and PD-nFOG during normal stepping using fNIRS, and meanwhile, in PD-nFOG patients, the duration of PD was positively correlated with the activation of PMC and SMC. Both their study and this present study revolve around the frontoparietal network, and it has been found that the functional connections in the motor integration brain regions of PD patients are enhanced. The specific brain regions involved in the two studies are similar. However, we added the PFC, which is crucial for motor planning and executive control, and singled out the PSC from their SMC. Another study (Alexandra et al., 2019) did not find any significant correlations between the measures of the severity of PD and the changes in brain FC within the cortico-basalganglia-thalamic circuitry. Compared with our study, in addition to the fact that they studied resting-state FC while our subjects were under the walking task, the cognitive level requirement in their study is a Montreal Cognitive Assessment (MoCA) score of 26 or more. However, we did not exclude subjects with mild cognitive impairment. The complex effects of cognitive abilities, especially executive functions, on the frontoparietal cortical neural network in PD cannot be denied. Besides, their study occurred in the ON-medication state for PD patients. These may explain part of the differences in the results of the two studies.

Interestingly, our study shows that the FC of brain regions related to gait speed and stride length is biased towards the right side. A previous study (Komal et al., 2019) also demonstrates that the rsFC between the right frontoparietal and executive-control RSNs negatively correlated with FOG-Q in PD-FOG patients. A possible explanation is as follows, studies have shown that when a task has higher requirements for attention, hemispheric asymmetry occurs. More specifically, in the parietal lobe, the visuospatial map of the right hemisphere changes from encoding only the visual targets on the contralateral side to encoding the memory and attention targets across the entire visual field (David and Summer, 2014). Therefore, for PD patients, when performing the task of walking which depends on strong spatial attention, the right frontoparietal lobe plays a more crucial role.

## 5 Limitations

Nevertheless, the limitations of the current study should be acknowledged. First of all, this is a cross-sectional study, and PD patients with Hoehn and Yahr stage IV were not included. These may have affected the generalizability of the results to the broader PD population. Secondly, this study only included the functional connections in the key frontal and parietal cortical regions related to walking. Given the roles of the occipital cortex in spatial perception, movement integration, and postural stability, it can be incorporated into future studies. Thirdly, our PD subjects were in the off-state of levodopa. Considering the individual variations in pharmacokinetics, and long-term effects of levodopa on gait and cortex, we plan to include newly diagnosed and drug-naive PD patients, and those in the on-phase of drugs (reporting the best motor state) in future studies to reduce bias.

## 6 Conclusion

Our study concentrates on the FC in crucial frontal and parietal cortical regions during the walking of PD patients in real-life situations by means of fNIRS. This is conducive to enhancing the understanding of the neural mechanisms underlying PD gait disorders. Our research indicates that strengthening the FC within RPMC and between RPMC and RPSC could be a potential target for future treatments of PD. It also suggests that further research on comparisons before and after using levodopa, rehabilitation, and neuromodulation is of great significance and may provide a broader impact.

## Data Availability

The original contributions presented in the study are included in the article/supplementary material, further inquiries can be directed to the corresponding authors.
